# Legionella maceachernii Pneumonia Presenting as a Cavitary Lesion in an Immunocompromised Patient

**DOI:** 10.7759/cureus.85289

**Published:** 2025-06-03

**Authors:** Liliana Franco, Vidhu Kariyawasam, Clay C Evans

**Affiliations:** 1 Infectious Diseases, University of Florida, Gainesville, USA

**Keywords:** atypical pneumonia, cell-free dna sequencing, immunocompromised, legionnaires' disease, pulmonary cavitation

## Abstract

*Legionella* is an intracellular, gram-negative bacterium known as a less common cause of pneumonia, which has become a public health concern in recent years due to increased incidence. *Legionella pneumophila* is recognized as the most common cause of Legionnaires' disease. Other *Legionella* species, including *Legionella maceachernii*, have been rarely described as causes of Legionnaires' disease. Moreover, cavitary lesions as radiological findings in patients with Legionnaires' disease are uncommon but have been reported, especially in immunocompromised patients. Although the gold standard for diagnosis is a positive culture, the need for special media and extended time for growth can overlook or delay diagnosis. Additionally, urine antigen testing will miss detection of species other than *Legionella pneumophila* serogroup 1. Molecular testing, although not routinely performed, is a promising tool for early detection and identification of other *Legionella* species. We present a case of an immunocompromised patient presenting with fevers and a cavitary lesion, diagnosed with *Legionella maceachernii* through cell-free DNA sequencing.

## Introduction

*Legionella *is a less common cause of community-acquired pneumonia known as Legionnaires' disease. *Legionella pneumophila* species from serogroup 1 are the most commonly known to cause the disease, although other species have been described as well [1, 2]. *Legionella maceachernii *has rarely been reported in the literature as a cause of pneumonia, often affecting immunocompromised patients with severe progression and high mortality [3]. Clinical manifestations of *Legionella* pneumonia include fever, cough, headaches, and fatigue. Patients may also have gastrointestinal or neurological manifestations [1]. Immunocompromised patients usually have a severe clinical presentation with more complications [1]. Cavitary pulmonary lesions, although rare, have been described as a radiologic finding in patients with *Legionella *in approximately 10% of immunocompromised patients [1, 4, 5]. The gold standard for diagnosis of *Legionella *pneumonia is culture of bronchoalveolar samples. Growth often can take three to five days or longer and requires special media such as buffered charcoal yeast extract (BCYE) agar. Moreover, species other than *Legionella pneumophila *serogroup 1 may be missed by the more commonly ordered urine antigen testing [6]. Delayed recognition and treatment of *Legionella *have been associated with a poorer prognosis [7]. Molecular testing is of great advantage for rapid diagnosis in patients with high suspicion for *Legionella *pneumonia caused by uncommon species [6]. We describe a case of an immunocompromised patient with a rare presentation of *Legionella maceachernii *with cavitary pulmonary lesions diagnosed through cell-free DNA sequencing.

## Case presentation

A 31-year-old female presented to the emergency department complaining of recurrent fever. The patient had a past medical history of subcutaneous panniculitis-like T-cell lymphoma diagnosed three years prior. She was initially treated with cyclosporine and prednisone 50 mg daily, but recent imaging showed progression of the disease. Cyclophosphamide, doxorubicin, vincristine, and prednisone (CHOP) chemotherapy was started, with her first cycle administered one week prior to presentation. The patient also received high-dose prednisone (100 mg daily) post-chemotherapy. The patient had first presented to the hospital five days prior to this admission with a fever of 103.3 °F, generalized fatigue, mild abdominal tenderness, and vomiting for one day. She was admitted for neutropenic fever, and infectious workup, including blood cultures, FLU/COVID-19/RSV (respiratory syncytial virus) testing, chest X-ray, and urinalysis, was negative. The patient's fever defervesced on cefepime after three days, and she was discharged.

She then returned to the hospital one day after discharge with complaints of recurrent fever, with a maximum temperature of 102 °F. The fever was associated with sore throat, mild productive cough, and fatigue. She denied chest pain, shortness of breath, nausea, vomiting, diarrhea, dysuria, or hematuria. Her vital signs were: blood pressure 105/73 mmHg, heart rate 142, respiratory rate 18, temperature 103.1 °F (39.5 °C), and oxygen saturation 100% on room air. Notable physical exam findings included: a clear oropharynx, no lymphadenopathy, tachycardia with a normal rhythm and no murmurs, clear lungs to auscultation with no wheezing, rhonchi, or rales, a non-tender abdomen with no hepatosplenomegaly, and no neurologic or notable skin findings. The patient’s previously noted leukopenia had improved, with neutropenia now resolved (Table 1). Initial workup showed negative COVID-19, influenza, and RSV testing. A pharyngeal *Streptococcus* Group A swab was negative. A BioFire® Respiratory Panel PCR was also negative. Chest X-ray was noted to be clear, with no consolidation.

**Table 1:** Laboratory findings WBC: white blood cell count, Hgb: hemoglobin, AST: aspartate transaminase, ALT: alanine transaminase, ANC: absolute neutrophil count.

Variables	Day 1 (first presentation to the hospital)	Day 2	Day 5 (current presentation)	Reference range
WBC	0.1	1.7	3.6	4.0–10.0 × 10^3^/µL
Hgb	8.8	8.7	8.4	12.0–16.0 g/dL
Platelet	120	105	296	150–450 × 10^3^/µL
Sodium	128	129	128	136–145 mmol/L
Serum creatinine	0.68	0.69	0.72	0.38–1.02 mg/dL
AST	34	NA	17	0–37 IU/L
ALT	52	NA	115	0–35 IU/L
ANC	None seen	1.28	3.20	1.70–7.00 x10^3^/µL

**Figure 1:**
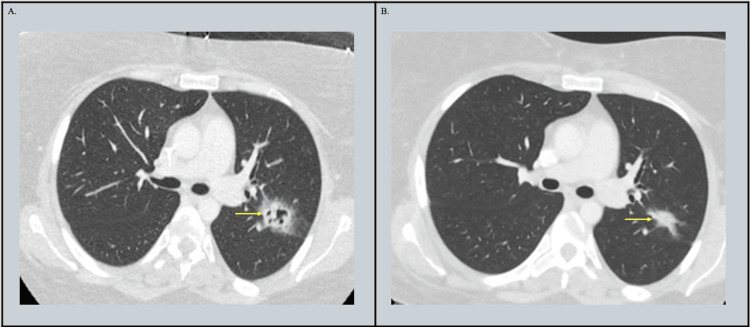
(A) Initial chest CT at admission showing cavitary lesion in the left upper lobe. (B) CT after 27 days of levofloxacin showing resolution of cavitation.

The patient was admitted for further evaluation and started on empiric cefepime and linezolid, though she continued to spike fevers. Chest computed tomography (CT) showed a 2.4 × 2.0 cm cavitary lesion in the apicoposterior segment of the left upper lobe with surrounding ground-glass opacification consistent with a halo sign. Radiology noted that the differential included angioinvasive aspergillosis, given that the patient was immunocompromised (Figure 1). Voriconazole was started empirically. Further workup included a serum *Aspergillus galactomannan* antigen, which was negative with an index of 0.7, and blood (1,3)-beta-D-glucan, which was also negative at 9 pg/mL. 

On hospital day 4, bronchoscopy with bronchoalveolar lavage (BAL) fluid analysis was obtained. Cell count was notable for 60% polymorphonuclear neutrophils (PMNs), 5% lymphocytes, and 35% monocytes. BAL cytology revealed no evidence of malignancy, and no fungal organisms (including *Pneumocystis jirovecii*) were identified on a GMS (Grocott’s methenamine silver) stain. No viral cytologic changes were identified. Flow cytometry noted no overt evidence of a clonal B-cell or aberrant T-cell population. Bacterial, fungal, and AFB cultures were negative. No special media cultures, including BCYE agar, were performed. Further testing was negative, including *P. jirovecii* PCR, BAL *Aspergillus galactomannan *antigen, urine *Histoplasma galactomannan *antigen, blood *Cryptococcus *antigen, *Histoplasma* serology, *Blastomyces *serology, and *Coccidioides *serology.

Despite empiric treatment and the above negative workup, the patient continued to have fevers above 102 °F daily. On hospital day 8, a next-generation sequencing (NGS) test for cell-free DNA (cfDNA) (Karius®) in the blood was ordered. On hospital day 10, the test returned positive for *Legionella maceachernii *with 155 molecules per 100 nanoliters, and the patient was switched to levofloxacin. The patient had notable clinical improvement with resolution of fevers over the following 24 hours. She was discharged on levofloxacin to complete a four-week course. A repeat CT scan four weeks later showed that the previously seen cavitary consolidation in the posterior left upper lobe had decreased in size and was no longer cavitary (2.1 × 1.3 cm, previously 2.4 × 2.0 cm) (Figure 1). Levofloxacin was extended for an additional two weeks to complete a six-week total course of treatment.

## Discussion

Legionellosis is an infection caused by an intracellular, gram-negative bacterium of the genus *Legionella*. The usual presentation is characterized by symptoms of atypical pneumonia, known as Legionnaires' disease, which is commonly caused by *Legionella pneumophila* species, though other species have also been reported [2]. Characteristics that increase risk include advanced age, chronic lung disease, male sex, immunosuppression, underlying malignancy, alcohol abuse, smoking, and use of anti-tumor necrosis factor (TNF)-alpha agents [1, 2]. *Legionella* is transmitted by aerosolized contaminated water from the environment or water system. The incidence of Legionnaires' disease has been increasing in the United States, with a 192% increase from 2000 to 2009, becoming a public health concern [1]. Despite this increase, it is often underdiagnosed or misdiagnosed. The most common symptoms include fever and cough associated with myalgias, headaches, anorexia, and pleuritic chest pain. Other associated symptoms that should prompt consideration of Legionnaires' disease include gastrointestinal symptoms (nausea, vomiting, diarrhea, and abdominal pain) and neurological symptoms (obtundation, seizures, and focal neurological symptoms) [1]. *Legionella maceachernii* has rarely been described as the cause of pneumonia. Currently, there are seven reported cases of pneumonia and one of skin and soft tissue infection caused by this species. Of the patients presenting with pneumonia, six were described as immunocompromised, and four patients died [3, 8].

The most common radiological findings in patients with *Legionella* pneumonia include multilobar pneumonia or multisegmented pulmonary infiltrates with airspace consolidations and ground-glass opacities [4]. Cavitary pulmonary lesions have been reported with Legionnaires' disease on rare occasions. In a retrospective review of CT scans, all 12 patients with *Legionella pneumophila* pneumonia and cavitary lesions were noted to be on high-dose steroids [4]. Moreover, reported cases in the literature of *Legionella pneumonia* with cavitary lesions were noted more often in patients with a suppressed immune system, including transplant patients [9], immunodeficiency syndrome [10], systemic lupus erythematosus [11], and AIDS [12]. *Legionella maceachernii* has also shown radiological findings with progressive cavitation, lung abscess formation, and hemorrhage [3, 13, 14]. Additionally, the radiological appearance of cavitary lesions can sometimes be misdiagnosed as invasive mold infection, especially in immunocompromised patients [5].

Diagnosis of Legionnaires' disease is a combination of a high index of clinical suspicion and confirmatory testing. Confirmatory diagnostic testing includes molecular testing, urine antigen tests, and culture. Culture is the gold standard test for identification of species and susceptibilities. Unfortunately, culture can be difficult to obtain due to a lack of growth, the need for special media (BCYE), and prolonged culturing time. These factors lead to a delay in diagnosis and prompt treatment [2]. In general, culture takes at least three to five days and up to two weeks to result [6]. Several cases of *L. maceachernii* were reported in the literature, and of those confirmed cases, some cultures were negative, while others were positive only after seven days of incubation [13, 14]. Another limitation to diagnosis is that many microbiology laboratories do not perform *Legionella* cultures. The fastest and most available diagnostic test is the urine *Legionella* antigen test, though this only accurately detects serogroup 1 *Legionella pneumophila* species, which causes 50-80% of cases of Legionnaires' disease. This limitation may lead to a false negative result in patients infected with other *Legionella *species, prompting inappropriate de-escalation of treatment [6]. In patients with severe community-acquired pneumonia, testing for other species should be considered. Molecular testing, including nucleic acid-based detection or PCR from bronchial and sputum samples, detects all species with high specificity and sensitivity, but has low sensitivity in serum [2]. This test also requires special technology that is not always readily available. DNA sequencing was used for diagnosis in several cases of *L. maceachernii* reported in the literature.

Most recently, cell-free DNA sequencing has led to increased diagnosis of *Legionella* pneumonia. Cell-free DNA sequencing is a noninvasive diagnostic test used to detect many organisms, including bacteria, fungi, DNA viruses, and eukaryotic parasites, using a single blood sample. It has been shown to be a helpful tool for diagnosis, especially in immunocompromised patients. In a recent retrospective study that looked into the use of cell-free NGS, 36 patients (92% immunocompromised) were evaluated, and in 52% of the cases, the use of cell-free DNA sequencing had a positive clinical impact, either establishing a new diagnosis or optimizing treatment [15]. In another retrospective study evaluating 82 cell-free DNA tests used in adults and children, a positive impact was noted in 7.3% of patients in whom conventional microbiology methods could not detect or confirm an organism [16]. Although routine use of this test needs further study, testing should be considered in certain clinical scenarios and patients.

Treatment for Legionnaires' disease should be promptly initiated if there is a high index of suspicion, as delays in treatment have been associated with poor prognosis and higher mortality. Current guidelines for community-acquired pneumonia recommend the use of fluoroquinolones or a macrolide for the treatment of Legionnaires' disease [7]. Recommendations for duration of treatment are five to seven days for mild disease, and an extended course is considered in immunocompromised patients, those with complications, or those with severe disease (10-14 days) [2]. A longer duration should be considered in patients with immunosuppression.

In our case, the patient had an underlying malignancy, received high-dose steroids before and after chemotherapy, and was neutropenic for a brief period, contributing to her immunocompromised state. Given her mild symptoms and radiological findings atypical of *Legionella* pneumonia, it was not initially considered in the differential. Cultures did not grow *Legionella*, and *Legionella *cultures are not routinely performed in our facility. Molecular testing, and in this case, cell-free DNA sequencing, proved to have a positive impact on the diagnosis and treatment of this patient. She recovered with an extended course of fluoroquinolones.

## Conclusions

*Legionella *as the cause of cavitary pneumonia is rare but should be included in the differential diagnosis, especially in patients with a compromised immune system. Although most cases of Legionnaires' disease are caused by *Legionella pneumophila *serogroup 1, other groups or species of *Legionella *should be considered, particularly when a patient presents with severe atypical pneumonia and there is a high index of clinical suspicion. In such cases, molecular testing and cell-free DNA sequencing may be beneficial for prompt diagnosis and to reduce treatment delays.
